# Supplementary material for: South African Healthcare Professionals’ Knowledge, Attitudes, and Practices Regarding Environmental Sustainability in Healthcare: A Mixed-Methods Study
Source: Int J Environ Res Public Health. 2022 Aug 16;19(16):10121. doi: 10.3390/ijerph191610121 (PMC9408692; doi:10.3390/ijerph191610121)
Supplement: Supplementary file 1 [file ijerph-19-10121-s001.zip › ijerph-1842400-supplementary.pdf]

International Journal of
Environmental Research and Public Health

MDPI

Supplementary Material

ANNEXURE S1: Knowledge, attitudes and practices (KAP) questionnaire

Please do not use without referencing the authors

SECTION 1

Participant Demographic Information (Section 1/5).
Do you reside in South Africa?

○ Yes

○ No

What health profession do you belong to?

○ Occupational Therapy

○ Physiotherapy

○ Speech-Language Pathology

○ Audiology

○ Both Speech-Language Pathology and Audiology

○ Dietetics / Human Nutrition

Participant's sex:

○ Male

○ Female

○ Other

○ Prefer not to say

Please state your age: ____

What region of the country are you currently primarily working in?

- ○ Northern Cape
- ○ Limpopo
- ○ Free State
- ○ Eastern Cape
- ○ Mpumalanga
- ○ KwaZulu-Natal
- ○ Western Cape
- ○ Gauteng
- ○ North West

What university did you obtain your undergraduate degree from?

- ○ University of Pretoria
- ○ University of Johannesburg
- ○ University of Witwatersrand
- ○ University of Cape Town
- ○ University of KwaZulu-Natal
- ○ Stellenbosch University
- ○ University of the Free State
- ○ University of the Western Cape
- ○ North-West University
- ○ Sefako Makgatho Health Sciences University
- ○ Other (please specify below)

If you selected 'other', please specify here: ____

What are your primary areas of work? (You may select more than one answer):

- ☐ Education
- ☐ Community
- ☐ Private practice
- ☐ Primary Healthcare
- ☐ Acute / Secondary Care or Rehabilitation
- ☐ Mental health services
- ☐ Academia
- ☐ Non-governmental Organization (NGO)
- ☐ Older persons residential facility
- ☐ Tertiary Healthcare
- ☐ Other (please specify below)

If you selected 'other', please specify here: ____

SECTION 2

Please select the answer that best describes your knowledge regarding environmental sustainability and healthcare practice (Section 2/5).

1. Please match the definitions you feel are most appropriate to the following terms:
(a) sustainable healthcare
(b) environmental sustainability
(c) environmental degradation.

Please use the drag (from the left column) and drop (into the right column) feature to do so.
Please look at the definitions below and drag the corresponding number to the column you feel is the most appropriate. Only one definition may be matched to each term.
Please do not use a Google search to help you, we are interested in your current knowledge.

Definition 1: “A process through which the natural environment is compromised in some way, reducing biological diversity and the general health of the environment. This process can be entirely natural in origin, or it can be accelerated or caused by human activities”.

Definition 2: “The use of our resources and environment to meet ‘the needs of the present generation without compromising the ability of future generations to meet their needs”.

Definition 3: “Care delivered in a way that does not affect the health of the population in an unfavourable manner and does not use resources in a way that might compromise the ability of those in the future to provide high quality care to their population or increase their burden of care”.

Definition 4: "The global phenomenon of climate transformation characterized by the changes in the usual climate of the planet (regarding temperature, precipitation, and wind) that are especially caused by human activities".

Definition 5: "The science and practice preventing human injury and illness, and promoting wellbeing by identifying and evaluating environmental sources and hazardous agents".

Definition 6: "The amount of information individuals have concerning environmental issues and their ability to understand and evaluate its impact on society and the environment".

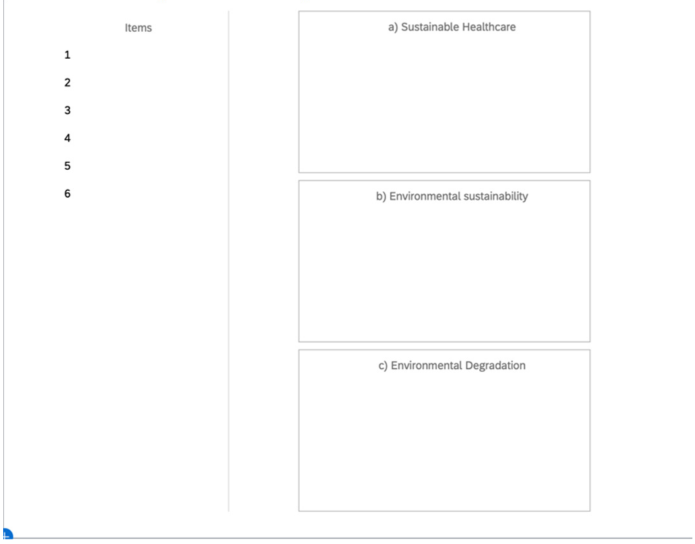

2. In practicing as a healthcare professional, do you consider environmental sustainability when using/purchasing equipment, resources, consumables, and/or devices?

○ Yes (please elaborate below)

○ No

If you selected 'Yes', please elaborate here: ____

3. What equipment, resources, consumables and/or devices that you use in your practice, do you think contributes to climate change and/or environmental degradation? If so, how? ____

4. How would you describe your knowledge on the following topics:

	Extensive	Good	Limited	No knowledge
Environmental Degradation	○	○	○	○
Environmental Sustainability	○	○	○	○
Sustainable Healthcare	○	○	○	○

5. Do you think environmental degradation affects disease patterns and human health?

○ Yes (please elaborate below)

○ No

○ I don't know

If you selected 'Yes', please elaborate here: ____

SECTION 3

Please select the answer that best describes your attitude regarding environmental sustainability and healthcare practices (Section 3/5).

On a scale of strongly disagree to strongly agree, please rate the following statements:

	Strongly Disagree	Disagree	Neither agree nor disagree	Agree	Strongly Agree
1. The healthcare sector is currently taking environmental sustainability into consideration in healthcare practices.	○	○	○	○	○
2. Environmental sustainability should be incorporated into healthcare practices.	○	○	○	○	○
3. Healthcare professionals should take a leading role in **advocating** for environmental sustainability in the healthcare sector.	○	○	○	○	○
4. Healthcare professionals should take a leading role in **implementing** environmental sustainability in the healthcare sector.	○	○	○	○	○
5. The lack of environmental sustainability in the healthcare sector has an impact on our environment.	○	○	○	○	○
6. Environmental degradation has an impact on the health of individuals throughout their lifetime.	○	○	○	○	○
7. The current state of our environment (including the rate of climate change) is concerning.	○	○	○	○	○

8. I am interested in learning how to implement environmental sustainability strategies in my healthcare practice.	○	○	○	○	○
9. The **government** (Department of Healthcare) has to take responsibility in terms of environmental sustainability in the healthcare sector.	○	○	○	○	○
10. **Healthcare professionals** have to take responsibility in terms of environmental sustainability in the healthcare sector.	○	○	○	○	○
11. **Leadership in communities** (counsellors, chiefs etc.) have to take responsibility in terms of environmental sustainability in the healthcare sector.	○	○	○	○	○
12. **Individuals** (clients) have to take responsibility in terms of environmental sustainability in the healthcare sector.	○	○	○	○	○

SECTION 4

Please answer the following questions on the use of environmental sustainability in your healthcare practice (Section 4/5).

1. Are you currently implementing any form of environmental sustainability into your practices as a healthcare professional?

- ◯ Yes
- ◯ No

2. Have you ever thought about including environmental sustainable practices in your practice?

- ◯ Yes
- ◯ No

3. How do you think you can practice environmental sustainability within your profession? _________________

4. Would you be interested in implementing strategies into your practice that can contribute to environmental sustainability?

- ◯ Yes
- ◯ No

SECTION 5

Please answer the following questions regarding barriers in implementing environmental sustainability and healthcare practice (Section 5/5).

1. Please select from the list below, what could be hindering you from implementing environmental sustainability into your practice (you may select more than one answer):

- ☐ Lack of finances
- ☐ Lack of resources (please elaborate below)
- ☐ Lack of knowledge
- ☐ It takes too much time
- ☐ It is too much extra effort
- ☐ I am not interested in having an environmental sustainable practice
- ☐ Other (please specify below)

If you selected 'lack of resources' or 'other', please elaborate/specify here: ________________________________

2. What do you believe is the biggest challenge to implement environmental sustainable strategies in healthcare?

____

3. Do you think educational input should be given within your profession regarding environmental sustainability?

- ○ Yes (please elaborate below)
- ○ No

If you selected 'Yes', on which level do you think educational input regarding environmental sustainability should be given (for example: undergraduate university training/post-graduate university training/CPD courses)?

____

4. What is your preferred method of being educated on a topic? (You may select more than one answer).

- ☐ Via email
- ☐ Via seminar/ webinar
- ☐ Via videos
- ☐ Workshop
- ☐ In a pamphlet/ booklet
- ☐ Other (please specify below)

If you selected 'other', please specify here:____

Thank you for participating in this questionnaire!

Please click on the option below to confirm you are ready to submit your responses, and then on the arrow below to submit.

If you have any additional questions, please send an email to Richelle on the following email address: u18057293@tuks.co.za

- ○ I am ready to submit my responses
